# Automated linguistic and acoustic measures of verbal fluency as neurocognitive markers of mild cognitive impairment

**DOI:** 10.1002/alz.086150

**Published:** 2025-01-09

**Authors:** Gonzalo Nicolás Pérez, Joaquín Ponferrada, Franco Javier Ferrante, Iván Caro, Joaquín Valdez Bisé, Joaquín Migeot, Alejandro Sosa Welford, Loreto Olavarria, Patricia Pelle, Andrea Slachevsky Chonchol, Luciana Ferrer, Adolfo M. Garcia

**Affiliations:** ^1^ Cognitive Neuroscience Center, University of San Andrés, Victoria, Buenos Aires Argentina; ^2^ Consejo Nacional de Investigaciones Científicas y Técnicas (CONICET), Buenos Aires Argentina; ^3^ Universidad de Buenos Aires, Buenos Aires Argentina; ^4^ Department of Psychiatry, School of Medicine, Pontificia Universidad Católica de Chile, Santiago Chile; ^5^ Latin American Brain Health Institute (BrainLat), Universidad Adolfo Ibañez, Santiago Chile; ^6^ Center for Social and Cognitive Neuroscience, Universidad Adolfo Ibáñez, Santiago Chile; ^7^ Latin American Brain Health Institute (BrainLat), Universidad Adolfo Ibáñez, Santiago Chile; ^8^ Memory and neuropsychiatry disorders Clinic (CMYN), Santiago Chile; ^9^ Latin American Brain Health Institute (BrainLat), Universidad Adolfo Ibanez, Santiago Chile; ^10^ Adolfo Ibañez University, Santiago, Region Metropolitana Chile; ^11^ Neuropsychology and Clinical Neuroscience Laboratory (LANNEC). Faculty of Medicine, University of Chile., Santiago, Region Metropolitana Chile; ^12^ Geroscience Center for Brain Health and Metabolism (GERO), Santiago Chile; ^13^ Instituto de Ciencias de la Computación, Universidad de Buenos Aires, Buenos Aires Argentina; ^14^ Universidad Santiago de Chile, Estación Central, Santiago de Chile Chile; ^15^ Global Brain Health Institute, University of California, San Francisco, CA USA

## Abstract

**Background:**

Verbal fluency tasks are routinely employed in screening for mild cognitive impairment (MCI). Yet, traditional outcome measures focus on the number of valid responses, failing to reveal which specific semantic memory dimensions may be altered and limiting analyses to univariate methods. Building on recent findings on Alzheimer’s disease, we employed automated methods to establish which linguistic and speech timing features better discriminate MCI patients from healthy controls (HCs), including machine learning analyses, comparisons with standard neuropsychological tests, and brain‐behavior correlations.

**Method:**

We recruited 106 native Spanish speakers (52 with MCI, 54 HCs), who completed phonemic and semantic fluency tasks as well as standard tests of attention (Trail Making Test‐A) and episodic memory (Free and Cued Selective Reminding Test). Responses in the fluency tasks were audio‐recorded and transcribed for automatic extraction of word properties (granularity, frequency, phonological neighborhood, word length, imageability, familiarity) and speech timing features (number of syllables, pauses, pause duration, phonation time, articulation rate, average syllable duration). These variables were compared between groups through a generalized linear model (GLM, with standard cognitive test scores as covariates) and fed into a binary classifier for subject‐level discrimination. Structural and functional brain measures were obtained from 63 participants and subjected to voxel‐based morphometry and seed‐to‐voxel resting‐state connectivity analyses. Correlations between behavioral and brain features were examined via multiple regressions.

**Result:**

GLM analysis revealed significant group differences in specific word properties (granularity, frequency, word length, imageability), but not in speech timing features. Subject‐level classification was better when based on word properties (AUC = 0.72) than on speech timing features (AUC = 0.63), with maximal discrimination upon combining both dimensions (AUC = 0.78). MCI patients exhibited atrophy left temporal atrophy and altered connectivity between the right parahippocampus and fronto‐posterior cortical regions. Word frequency was negatively correlated with insular volume and granularity was positively correlated with the volume of fusiform and parahippocampal regions.

**Conclusion:**

Automated analyses of word properties and timing features in verbal fluency tasks offer novel insights into cognitive decline, surpassing traditional tests in identifying MCI. Their widespread implementation could inaugurate a promising avenue to establish scalable markers of the condition.

